# Seed Coatings with Melatonin-Embedded Hydrogel Biopolymers as Green Tools to Mitigate Salinity Stress in Tomato Plants

**DOI:** 10.1021/acsapm.5c01261

**Published:** 2025-07-31

**Authors:** Kyriakos Athanasiou, Andreas Ioannou, Egli C. Georgiadou, Alice Varaldo, Evangelia Tarani, Konstantinos Chrissafis, Vasileios Fotopoulos, Theodora Krasia-Christoforou

**Affiliations:** † Department of Mechanical and Manufacturing Engineering, 54557 University of Cyprus, 1 Panepistimiou Avenue, Aglantzia, Nicosia 2109, Cyprus; ‡ Department of Agricultural Sciences, Biotechnology & Food Science, 121991 Cyprus University of Technology, Limassol 3603, Cyprus; § Department of Agricultural, Food and Forest Sciences, 9314 University of Turin, Turin 10124, Italy; ∥ Laboratory of Advanced Materials & Devices, School of Physics, 37782 Aristotle University of Thessaloniki, Thessaloniki GR 54124, Greece

**Keywords:** alginate, hydrogels, melatonin, biostimulants, abiotic stress

## Abstract

Improving plant tolerance against abiotic stress factors is important for sustaining global food security. Plants show prolonged, increased tolerance upon exposure to suboptimal environmental conditions when treated at the seed stage with certain chemical agents of natural or synthetic origin, resulting in higher crop yields. Polymers such as naturally derived hydrogels (HYDR) provide a smart delivery system for phytohormones. The protective effect of melatonin (Mel) and FOLICIST (Fol) applied directly or as calcium alginate-based hydrogel coatings with embedded Mel (HYDR-Mel) was studied in the “Dafni F1” tomato cultivar against salinity stress under in vitro conditions. An array of agronomic and biochemical parameters was evaluated, further to detailed material characterization, including germination-related parameters (such as the germination rate index, mean germination rate, speed of germination, and emergence index), morphological alternations (such as the main root length and root fresh weight), as well as cellular damage indicators (such as malondialdehyde (MDA) and H_2_O_2_ content). Findings suggest that engineered HYDR-Mel formulations are a promising “green” approach to mitigate abiotic stress effects in plants.

## Graphical abstract

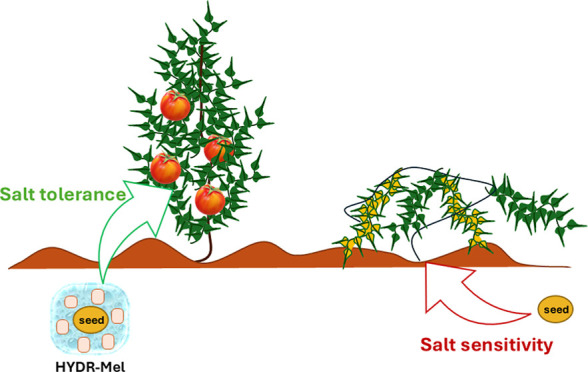

## 1 Introduction

Salinity in soil is one of the major abiotic stress factors that reduces crop productivity and yield worldwide. High salt concentration in the soil reduces the ability of the plant to absorb water, leading to osmotic stress and ion toxicity due to the excessive accumulation of Cl^–^ and Na^+^ and the replacement of K^+^ in key biochemical reactions.^1^ That influences photosynthesis and nutrient uptake and thus directly affects the normal growth and production of plants.^2^ Furthermore, it increases reactive oxygen species (ROS) formation and triggers oxidative stress, which causes significant damage to membranes and other important cellular structures.^3^ The most critical stages in the development of crops are seed sprouting and seedling development, which are the most vulnerable stages impacted by abiotic stresses like salinity. This stressor disturbs the water absorption of plants, causing delays and reductions in seed germination and normal seedling and vegetative growth. Plants under such conditions try to adapt through various biochemical and physiological strategies in order to restore ion and water imbalance.^4^

In the previous decades, these strategies were characterized and used for the development of more tolerant varieties through conventional breeding or genetic modifications.^1^ For example, the tomato ( L.) is among the most important model crops with high economic value, especially valuable to study salt tolerance due to its well-characterized genetics and convenient transforming techniques.^5^ Despite its broad adaptability and spreading, tomato yield has been reduced due to increased soil or irrigation water salinity. Some tomato cultivars have the genetic capacity to tolerate mild salt stress.^6^ However, higher salt concentrations negatively impact even these cultivars by inhibiting the important early-stage seed germination and seedling growth and causing later leaf growth reduction due to decreased cellular turgor, underperformed photosynthesis, and altered metabolic signaling.^7^ Therefore, it is essential to develop effective, low-cost methods for promoting seed germination and late tolerance to saline conditions, which are particularly vital for agricultural productivity in sodic soil. At present, seed priming and seed coating are two simple and low-risk alternative approaches for improving plant abiotic stress tolerance such as salinity.^8,9^

Seed priming involves treating seeds with natural or synthetic compounds before germination to induce a specific physiological state. This process acts as a signaling mechanism that triggers stress response pathways in the seeds, enabling plants to respond more quickly and efficiently when exposed to environmental stresses. This enhanced readiness to activate defense responses is known as the primed state.^10^ Seed priming helps plants respond to various stresses by regulating internal levels of different hormones, antioxidant enzymatic activities, and various molecular biological processes.^11^ More specifically, it can trigger metabolic activities, cell division, protein synthesis, and antioxidant activity, facilitating proper germination and seedling development and enabling them to overcome environmental stresses.^12–14^ The various approaches include hydropriming, osmopriming, chemical priming, hormonal priming, biological priming, redox priming, etc. Although priming improves the rate and uniformity of seedling emergence and growth, particularly under stress conditions,^15^ the effectiveness of different priming agents varies under variable stress conditions and with different crop species.^16^ Several types of naturally occurring molecules have been proposed as priming agents, including water; amino acids like proline;^17^ hormones like melatonin;^18^ reactive oxygen (e.g., H_2_O_2_), nitrogen (e.g., NO), and sulfur (e.g., H_2_S) species;^19^ polyamines;^20^ and more.

Melatonin (*N*-acetyl-5-methoxytryptamine) is an endogenous, naturally occurring indoleamine widely found in living, evolutionarily distant organisms. It was first isolated and extracted from bovine pineal glands.^21^ In animals, it acts as a neurohormone, having multiple effects on biological processes including circadian rhythms like sleep–wake cycles, mood, motor activity, body temperature changes, and reproductive physiology.^22,23^ Furthermore, it has anti-inflammatory and antioxidant effects.^24,25^ The wide range of its biological activities in plants has rightly implicated melatonin as being considered a multiregulatory molecule and suggested the use of the name “phytomelatonin”.^26^ It has been reported that melatonin can regulate plant development, including photosynthetic efficiency, biomass yield, seed germination, root elongation, leaf senescence, flowering, and ripening of fruits,^27,28^ in addition to acting as a protecting agent against abiotic and biotic stressors.^29–31^ Melatonin’s amphiphilic nature allows it to pass through different plant organelles and cellular structures, protecting them from oxidative damage by directly acting as a scavenger of reactive oxygen species (ROS) and reactive nitrogen species (RNS), reducing oxidative stress, and protecting cellular components. In addition, it can promote the activity of several antioxidant enzymes and defensive mechanisms, helping plants to withstand abiotic stresses.^32,33^ Previous studies have shown that cotton ( L.) seedlings that were primed at seed level with melatonin showed better photosynthetic efficiency and a stronger ability to scavenge ROS to salt stress,^34^ while similar findings were observed in peanut seeds primed with melatonin and subsequently submitted to drought stress conditions.^35^ Seed priming with melatonin reduced the negative effects of salinity on germination of and halophytes by improving mean final germination and rate of germination.^18^ Furthermore, similar growth parameters of zinnia plants () were improved under salinity stress. In addition, photosynthesis pigment contents were increased, while the activity of several antioxidant enzymes, such as superoxide dismutase (SOD), peroxidase (POD), and catalase (CAT), appeared to be enhanced, alleviating the effects of salinity stress. Hydrogen peroxide (H_2_O_2_) and malondialdehyde (MDA) levels recorded a decrease, while the osmoprotectants proline and glycine betaine had a significant increase.^36^

Functionalized seed coating technology (including covalent and noncovalent functionalization) has surfaced as a promising low-cost alternative method for plant abiotic stress protection. By providing a controlled slow release of active ingredients, nanomaterials and biopolymers ensure that seeds receive vital nutrients over a prolonged period while promoting improved plant development.^37^ Nanomaterials and biopolymers provide a smart delivery system for an array of priming agents, such as biostimulants, nutrients, hormones, and enzymes, that have the ability to enhance the half-life of these agents and improve crop growth by enabling a systematic release mechanism.^9,38^

Hydrogels are either physically or chemically cross-linked 3D polymer structures capable of absorbing high amounts of water without being dissolved. Their swelling behavior is governed by their architecture, chemical composition, and cross-linking density.^39^ Among those, naturally derived hydrogels based on collagen, chitosan, alginate, gelatin, elastin, peptides, etc., attract significant attention nowadays, especially in the biomedical field,^40^ due to their cost-effectiveness and biocompatibility.

Among the materials used in the preparation of naturally derived hydrogels, alginate—that is, a natural, linear, copolymer consisting of β-d-mannuronic acid (M) and α-l-guluronic acid (G), mainly produced from brown algae^41^—has been widely used due to its biocompatibility, biodegradability, lack of toxicity, cost-effectiveness, and facile gelation. Regarding the latter, alginate undergoes a rapid gelation/cross-linking process in the presence of divalent cations such as Ca^2+^.

In addition to the extensive applicability of naturally derived hydrogels in the biomedical field, their tunable mechanical properties and biodegradability profile, along with their ability to retain high amounts of water and nutrients within their 3D structure and release them in a sustainable manner, thus endorsing a drought resistance and plant growth promotion effect, respectively, enabled their further exploitation in the agricultural sector.^42–45^

Herein, alginate-based hydrogels (HYDR) of various cross-linking densities were synthesized and characterized with respect to their morphology, swelling behavior, and thermal and mechanical performance. The incorporation of melatonin (Mel) within the HYDR was carried out by means of physical entrapment in order to investigate its effect on tomato responses (“Dafni F1” tomato cultivar) against salinity stress under an in vitro experimental setup. Through the comprehensive evaluation of diverse agronomic and biochemical parameters, the HYDR-Mel formulations demonstrated marked protective effects against salinity-induced damage. These findings highlight a promising, ecofriendly strategy to mitigate abiotic stress in crops. Importantly, this innovative seed coating technology opens new avenues for sustainable agriculture, warranting further investigation into its underlying mechanisms.

## 2 Materials and Methods

### 2.1 Materials

Alginic acid sodium salt from brown algae (A2033, molar mass = 21,900 g·mol^–1^ mannuronate residues to guluronate residues (M/G) ratio = 2.12)^46^ and melatonin (*N*-acetyl-5-methoxytryptamine; Sigma-Aldrich Co., LLC., CAS number: 73-31-4 powder, ≥98% (TLC)) were purchased from Sigma-Aldrich. Calcium chloride anhydrous (97%) was supplied by HiMedia Laboratories. In all experiments, deionized (DI) water was used in solution preparation. FOLICIST, a commercially available biostimulant, was purchased from Biolchim SpA, Italy. All reagents were used as received from the manufacturer without further purification.

### 2.2 Material Synthesis and Preparation of Plant Treatments

#### 2.2.1 Alginate Hydrogels (HYDR)

All experiments were carried out under ambient conditions. For the preparation of alginate hydrogels of various cross-linking densities, the amount of sodium alginate (SA) was retained the same, and only the amount of CaCl_2_ (cross-linker) varied. In a typical process, sodium alginate (50 mg) was placed in a 15 mL glass vial together with deionized water (4 mL), and it was dissolved upon stirring for 2 min using a vortex mixer. CaCl_2_ (20 mg) was weighed in a separate vial. Deionized (DI) water (1 mL) was then added, resulting in complete dissolution of the salt. Subsequently, the two aqueous solutions were mixed and stirred using a vortex mixer for 20 s, resulting in the formation of the alginate hydrogel (HYDR1). Following the same process, 2 more HYDR were prepared by varying the amount of CaCl_2_ (i.e., 30 mg and 50 mg CaCl_2_, respectively). The produced hydrogels (HYDR2 and HYDR3, respectively) were then placed in DI water for 7 days to reach their equilibrium swelling state.

#### 2.2.2 Alginate/Melatonin Hydrogels (HYDR2-Mel)

HYDR with embedded Mel (denoted as HYDR2-Mel) was prepared as follows: melatonin (23 mg) was dissolved in DI water (20 mL) using a vortex mixer. A 16 mL portion of the resulting Mel solution was added into a glass vial containing sodium alginate (200 mg), while the remaining 4 mL was used to dissolve CaCl_2_ (120 mg). Subsequently, 4 mL of the sodium alginate/Mel aqueous solution was mixed together with 1 mL of the CaCl_2_/Mel aqueous solution for 20 s using a vortex mixer, resulting in the formation of the HYDR2-Mel.

#### 2.2.3 Fabrication of HYDR2-Mel Seed Coatings

HYDR2-Mel seed coatings were prepared as follows: melatonin/alginate and melatonin/CaCl_2_ solutions were prepared as described in part 2.2.2. Seeds were placed on a hydrophobic surface (Petri dish covered with parafilm). By using a micropipette, 16 μL of melatonin/alginate solution was placed on top of each seed, forming a droplet that surrounded the seeds. Subsequently, 4 μL of melatonin/CaCl_2_ was placed on top of each droplet, resulting in the instant formation of HYDR2-Mel seed coatings.

#### 2.2.4 Seed Treatments

Tomato seeds ( L. cultivar “-Dafni F1”) were surface-disinfected with 70% ethanol for 3 min, followed by 12 min incubation with 2% sodium hypochlorite, and then rinsed at least 5 times with sterile distilled water. Subsequently, a total of 720 seeds were split into 6 different groups, corresponding to the treatments of the experiment presented in detail in Table 1. An untreated group served as the main control. Hydroprimed seeds represent solvent control samples, while FOLICIST (Biolchim SpA, Italy) is a commercially available biostimulant that was employed as the positive control.

**Table 1:** Various Treatments Employed in the Designed Experimental Setup

treatment abbreviation	treatment
UNT	untreated (control)
HYD	hydroprimed (water control)
MEL	melatonin
HYDR	hydrogel (control)
HYDR-MEL	hydrogel/melatonin
FOL	FOLICIST

Seed priming was performed by soaking tomato seeds in each solution for 12 h at 25 °C in the dark. Then, the seeds were placed in gauze under the laminar flow for air drying until reaching their initial weight. Hydroprimed stands for normal distilled water. Melatonin (*N*-acetyl-5-methoxytryptamine; Sigma-Aldrich Co., LLC., CAS number: 73-31-4) was prepared in a stock solution of 200 μM using warm water under constant stirring for 20 min at 55 °C. The working solutions were prepared by diluting the stock in distilled water to reach a concentration of 50 μM. Finally, FOLICIST was diluted in 0.15% (v/v) H_2_O according to the manufacturer’s instructions.

For the production of seed coating hydrogels, 50 mg of sodium alginate and 30 mg of calcium chloride were diluted in 4 and 1 mL of water, respectively. For hydrogel/melatonin conjugates, these two chemicals were dissolved in 50 μM melatonin solutions. Each gel was produced by soaking each seed in 16 μL of sodium alginate solution, followed by the addition of 4 μL of calcium chloride, resulting in instantaneous gelation and seed encapsulation within the melatonin-containing hydrogel coating. The seeds were placed on a Petri dish under a laminar flow to air-dry for approximately 1 h. Final concentrations were chosen after screening in preliminary experiments (data not shown).

### 2.3 Materials Characterization

#### 2.3.1 Morphological and Chemical Characterization

The morphology of the produced hydrogels was investigated by scanning electron microscopy (SEM) (Vega TS5136LS-Tescan). Prior to visualization, the water-swollen hydrogels were lyophilized using a lyophilizer (Christ Alpha 1-2 LDplus; Martin Christ, Osterode, Germany) and Au-sputtered (Au film thickness: 30 nm) using the K575X Turbo Sputter Coater-Emitech sputtering system. The chemical composition of the produced HYDR was verified by FTIR on a Jasco FTIR 4100 spectrometer. The samples were ground with KBr to form semitransparent pellets that were inserted in the FTIR spectrometer for analysis.

#### 2.3.2 Swelling Behavior

After being immersed in deionized water for 1 week, thus reaching their swelling equilibrium state, the water-swollen hydrogels having a variable Ca^2+^ content (denoted as HYDR1, HYDR2, and HYDR3) were cut into small pieces (4× per hydrogel type), and their water-swollen mass was determined gravimetrically using a high-precision balance. Subsequently, all samples were placed in a vacuum oven at 40 °C for 6–8 h to dry, and their dry mass was determined gravimetrically. The degrees of swelling (DS) were determined as the ratio of the swollen mass to the dry mass.

#### 2.3.3 Thermal Characterization

Thermogravimetric analysis (TGA) of the hydrogels was performed by using a Labsys evo TGA/differential scanning calorimetry (DSC) 1150 SETARAM system (Caluire, France). The samples were heated from 25 to 500 °C under a nitrogen flow rate of 50 mL/min, with a heating rate of 10 °C/min. Each sample (24 ± 0.5 mg) was placed in an alumina crucible, and an empty alumina crucible was used as a reference. For the TGA analysis of melatonin, the temperature range was extended from room temperature to 700 °C, with the same heating rate of 10 °C/min. Continuous measurements of sample temperature, weight, the first derivative of weight, and heat flow were recorded throughout the experiment.

#### 2.3.4 Mechanical Properties

Unconfined compression tests were performed on the water-swollen HYDR instrument on a mechanical testing system (Instron 5944, Norwood, MA, USA). More precisely, at least 2 specimens from each sample (dimensions: 4.2 mm × 5.6 mm × 6.3 mm, height × width × thickness, respectively) were analyzed. The specimens were compressed to 30% strain with a strain rate of 0.1 mm min^–1^. The Young’s modulus was determined from the slope of the linear part of the stress–strain curves.

#### 2.3.5 Kinetic Release Studies

Kinetic release studies were performed by means of UV–vis spectrophotometry upon immersing the HYDR2-Mel-containing hydrogels (swollen mass: 2.34 g) in deionized water (3 mL) and recording the UV–vis spectrum of the supernatant solution between 200 and 400 nm at different time intervals, ranging from 0 to 180 min.

### 2.4 Plant Experimental Design and Growth Conditions

After finishing the treatments, 120 seeds for each treatment were divided into three different stress treatments as follows: (a) control with 0 mM of NaCl, (b) low to moderate with 50 mM of NaCl, and (c) moderate to severe with 75 mM of NaCl.^47^ These 40 seeds were split and placed in three Petri dishes (15 cm diameter) on double 125 mm Whatman filter paper with 8 mL of each solution sealed with parafilm and left to germinate in a versatile environmental test chamber (SANYO MLR-351) under controlled conditions (16 h light/8 h dark, 25 °C/22 °C, 150 μmol m^–2^ s^–1^). During the first 3 days, seeds remained in the dark.

### 2.5 Germination Index and Main Growth Parameters

The germination rate of the seeds was monitored twice daily at a specific time (10:00 and 18:00) for the entire duration of the 17 day experiment. On the last day of the experiment, the fresh weight of the seedlings was measured. Their length was photographed and analyzed later using the open-source software ImageJ. Finally, the seedlings of each treatment were pooled in three replicates, frozen with liquid nitrogen, and stored at −80 °C until further use. Eight different germination indicators were calculated based on the daily measurements from the first successful count (3rd day) until the end of the experiment (17th day), as appearing in the following:^48–50^

#### 2.5.1 Seed Germination Percentage (*G*)

It is the % of seeds that successfully germinate out of the total number of seeds sown.

1 $$G=\left[\sum \left(N_{i}\right)/k\right]\times 100\;\left(\%\right)$$

#### 2.5.2 Germination Rate Index (GRI)

The rate of germination over time took into account the number of seeds that germinated on each day.

2 $$GRI=\sum \left(N_{i}/i\right)\;\left(\%\;{day}^{-1}\right)$$

#### 2.5.3 Mean Germination Time (MGT)

The average time it takes for seeds to germinate gives an indication of the uniformity of the germination process.

3 $$MGT=\sum \left(N_{i}T_{i}\right)/\sum \left(N_{i}\right)\;\left(d\right)$$

#### 2.5.4 Coefficient of Velocity of Germination (CVG)

It indicates the speed at which seeds germinate, with higher values representing faster germination.

4 $$CVG=\left[\sum \left(N_{i}\right)/\sum \left(N_{i}T_{i}\right)\right]\times 100\;\left(\%\;{day}^{-1}\right)$$

#### 2.5.5 Coefficient of Variation of the Germination Time (CV)

It is a measure of the spread of germination times relative to the mean, expressed as a %.

$${CV}_{t}=S_{t}/t\times 100(\%)$$ 5

#### 2.5.6 Mean Germination Rate (MGR)

The inverse of the mean germination time (MGT) indicates the average rate at which seeds germinate.

$$MGR=1/\mathrm{MGT}\left({\mathrm{day}}^{-1}\right)$$ 6

#### 2.5.7 Uncertainty of the Germination Process (*U*)

A measure of the unpredictability or randomness in the germination process is expressed in bits.

$$U=-\overset{d}{\underset{i=1}{\sum }}\frac{N_{i}}{\overset{d}{\underset{j=1}{\sum }}N_{j}}\times \log_{2}\left(\frac{N_{i}}{\overset{d}{\underset{j=1}{\sum }}N_{j}}\right)\left(\mathrm{bit}\right)$$ 7

#### 2.5.8 Synchrony of Germination (*Z*)

It is a dimensionless measure of the synchronization of germination events among the seeds.

$$Z=\frac{\overset{d}{\underset{i=1}{\sum }}\frac{N_{i}(N_{i}-1)}{2}}{\frac{G(G-1)}{2}}\left(\mathrm{unit}\;\mathrm{less}\right)$$ 8

where Σ: summation symbol; *i*: day of observation; *t*: mean germination time; *G*: total number of germinated seeds; *N*_*i*_: number of seeds (*N*) germinated on day *i*; *T*_*i*_: time in days since sowing to day *i*; *k*: total number of seeds sowed for a replication of a treatment; *S*_*t*_: the standard deviation of germination time; and (d): days.

### 2.6 Determination of H_2_O_2_ Content

Plant hydrogen peroxide (H_2_O_2_) content was determined spectrophotometrically using potassium iodide (KI) as previously described by Loreto.^51^ After homogenizing 0.1 g of tissue with 1.5 mL of trichloroacetic acid (TCA; 0.1%), samples were centrifuged at 12,000*g* rpm at 4 °C for 20 min. The supernatant was used in 75 μL volume together with 75 μL of 10 mM potassium phosphate buffer (pH 7.0) and 150 μL of 1 M potassium iodide (KI) for the determination of H_2_O_2_. The absorbance of samples was determined spectrophotometrically at 390 nm in a 96-well plate with the Tecan Infinite 200 PRO plate reader (Tecan Trading AG, Switzerland). Levels of H_2_O_2_ were calculated using a standard curve prepared from H_2_O_2_ stock solution.

### 2.7 Determination of MDA Content

Lipid peroxidation levels, as a widely used cellular damage indicator, were determined from the quantification of malondialdehyde (MDA) content resulting from the thiobarbituric acid reaction, as previously described.^52^ The extraction method was identical to the H_2_O_2_ method as described previously. Then, 0.5 mL of supernatant was added to 1.5 mL of thiobarbituric acid (TBA) dissolved in 20% TCA. These mixtures were incubated at 95 °C for 25 min and then cooled on ice for at least 15 min. The absorbance of samples was measured using a spectrophotometer (Infinite 200 Tecan plate reader) at 532 nm and at 600 nm. MDA was quantified using an extinction coefficient of 155 mM^–1^ cm^–1^.

### 2.8 Statistical Analysis

For physiological and biochemical analyses, two-way ANOVA analysis was performed, followed by the Tukey-HSD post hoc test (*p* ≤ 0.05) using GraphPad version 9.4.0 (GraphPad Software, San Diego, CA, USA). Computations and figures were performed by GraphPad ver. 9.4.0 (GraphPad Software, San Diego, CA, USA).

## 3 Results and Discussion

### 3.1 HYDR Fabrication and Characterization

Following a physical cross-linking process, a series of HYDR in which the sodium alginate (SA) content was retained the same and only the amount of the ionic cross-linker (Ca^2+^) varied was synthesized as schematically depicted in Figure 1. The same figure presents schematically the fabrication concept of the present study, involving the generation of HYDR seed coatings with embedded Mel. The cross-linking process took place instantaneously by simply mixing the Ca^2+^ and SA aqueous solutions due to the development of electrostatic attractive forces between the hydroxyl and carboxylic functionalities that are found in the SA linear polymer chains and the Ca^2+^ ions.

**Figure 1:**
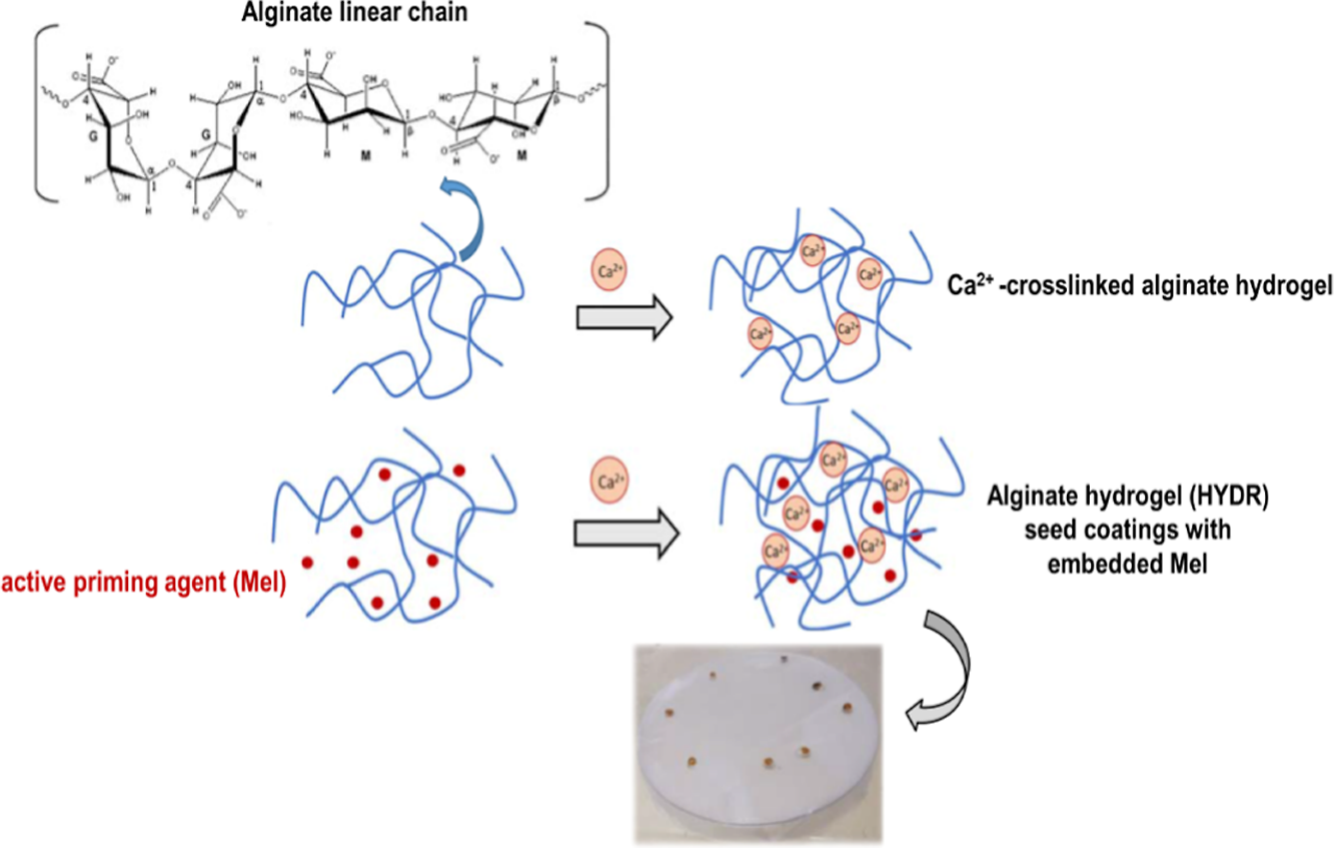
Schematic representation of the physical cross-linking process taking place between linear SA chains and Ca^2+^ ions, with the latter serving as the cross-linking agents. The same process applies for obtaining HYDR-Mel seed coatings.

FTIR spectroscopy was employed to verify the chemical composition of the produced HYDR. Exemplarily, the FTIR spectrum of HYDR1 is provided in Figure 2. The stretching vibrations of the O–H bonds of alginate appear within 3000 and 3600 cm^–1^, in line with previous findings,^15^ while stretching vibrations of aliphatic C–H are observed at 2920–2850 cm^–1^. The bands appearing around 1630 cm^–1^ can be attributed to the carboxylate ion, the one at 1425 cm^–1^ to the asymmetric and symmetric stretching vibrations, and those at 1100 and 1030 cm^–1^ to the C–O stretching vibration of the ring and the C–O stretching with contributions from C–C–H and C–O–H deformation, respectively. Additionally, bands appearing around 800 and 700 cm^–1^ can be assigned to mannuronic and guluronic acids, respectively, which are both present within the alginate structure, in line with previous reports.^16^

**Figure 2:**
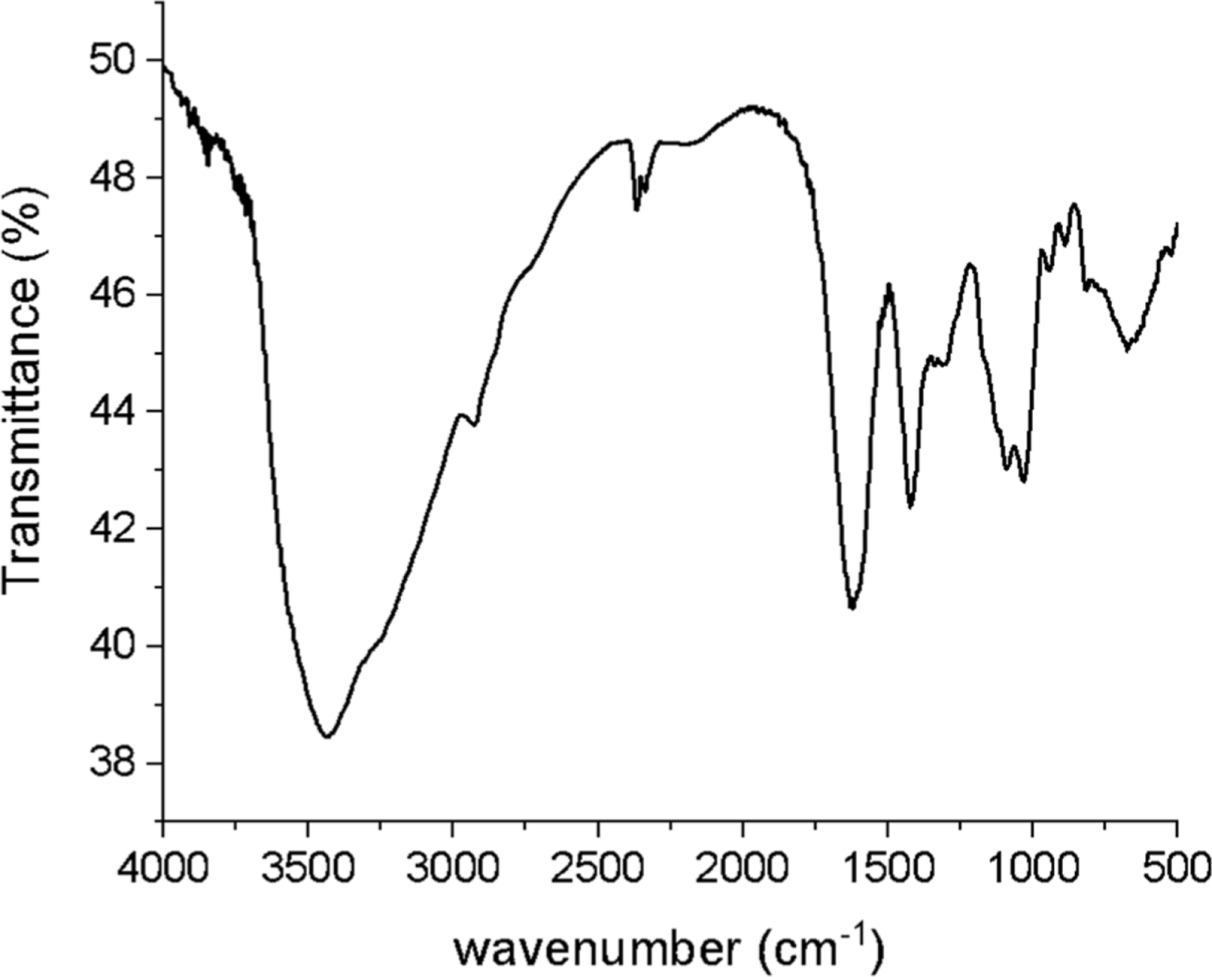
FTIR spectrum of HYDR1.

The swelling behavior of the synthesized HYDR was evaluated in deionized (DI) water. As seen in Table 2, the ability of the HYDR to retain water is reduced upon increasing the Ca^2+^ content and hence the HYDR cross-linking density.

**Table 2:** Swelling Ratio and Standard Deviation (SD) (Recorded in DI Water) of the HYDR with Variable Ca^2+^ Content Employed during Synthesis While Retaining the Amount of SA Constant (50 mg) in All Cases

HYDR	Ca^2+^ (mg)	swelling ratio and SD
HYDR1	20	41 ± 0.6
HYDR2	30	35 ± 1.5
HYDR3	50	30 ± 1.0

The morphological characteristics of the produced HYDR were investigated using scanning electron microscopy (SEM), and characteristic SEM images are provided in Figure 3.

**Figure 3:**
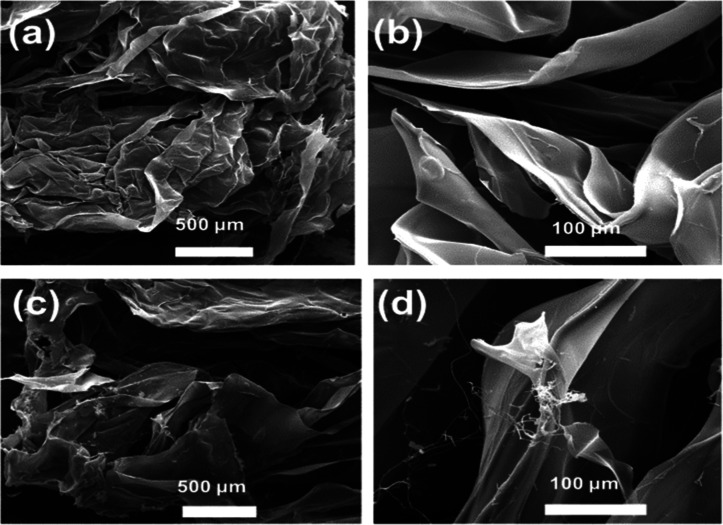
Representative SEM images of HYDR with the lowest Ca^2+^ content (20 mg, HYDR1) (a,b) and of the HYDR with the highest Ca^2+^ content (50 mg, HYDR3) (c,d).

A similar (highly porous) morphology was also observed in a recent study by our group dealing with the fabrication of 3D composites based on alginate hydrogels and biochar that were further employed as adsorbents for the removal of U­(VI) from aqueous media.^53^

The swelling behavior of the synthesized alginate hydrogels (HYDR1, HYDR2, and HYDR3) was further corroborated by thermogravimetric analysis (TGA) performed after their immersion in DI water for 7 days to reach their equilibrium swelling state. In the TGA plot of Figure 4, the thermal analysis of the equilibrated hydrogels (HYDR1, HYDR2, and HYDR3) shows that the initial mass loss below ∼150 °C corresponds to the evaporation of retained water, while the decomposition step between ∼170 and 300 °C is attributed to thermal degradation of the alginate network.^54^ The HeatFlow and the first derivative of the TGA (dTG) curves, shown as an inset in Figure 4b, provide verification of this, exhibiting endothermic and mass loss signals in the same temperature range. The mass loss was taken up to 150 °C (evaporation of free and bound water) and quantified to theoretically evaluate the water content in the swollen hydrogel.^55^ Values obtained were HYDR1: 82.5%, HYDR2: 73.0%, and HYDR3: 71.6%. It was found that the swelling ability of the synthesized alginate hydrogels is strongly dependent on the cross-linking density, which varies with the Ca^2+^ concentration. HYDR1 exhibited the highest water uptake, consistent with the highest swelling ratio, as it appears in Table 2. In contrast, HYDR3, prepared with the highest Ca^2+^ concentration, showed reduced swelling, confirming the effectiveness of ionic cross-linking in regulating the hydrogel network properties. Skopinska-Wisniewska et al.^55^ showed that increasing CaCl_2_ concentration reduces the swelling ability of alginate hydrogels due to higher cross-linking density.

**Figure 4:**
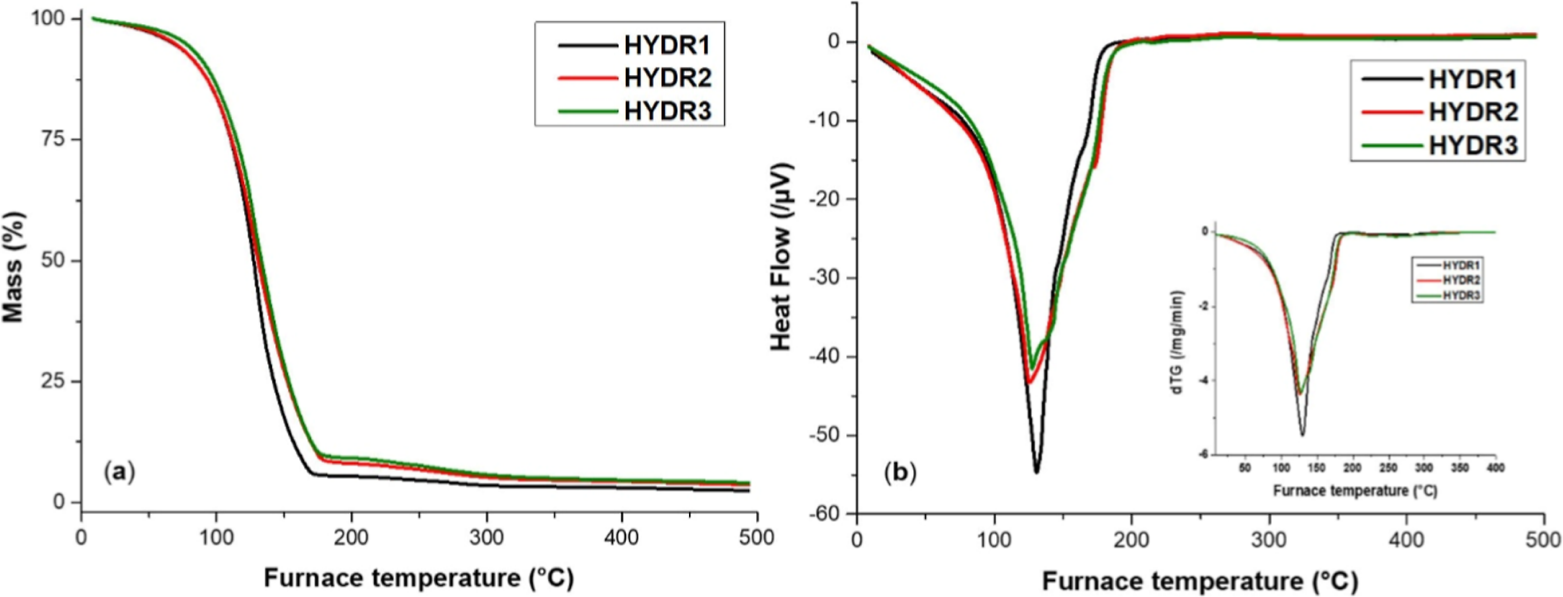
Thermal behavior of alginate-based hydrogels (HYDR1, HYDR2, and HYDR3) after 1 week of equilibration in water. TGA (a) and HeatFlow and dTG (inset) (b) curves.

The mechanical behavior of the synthesized HYDR was studied under unconfined compression on water-swollen specimens, and the corresponding stress–strain curves are provided in Figure 5. As seen, an increase in the Ca^2+^ content and thus in the cross-linking density leads to an increased stiffness and consequently a higher Young’s modulus, i.e., 2.89 ± 0.11 kPa compared to 3.90 ± 0.18 kPa corresponding to HYDR1 and HYDR3, respectively. These findings agree with the data related to the swelling behavior of the synthesized HYDR, since HYDR3, exhibiting the highest cross-linking density, is characterized by the lowest ability to swell in water and the highest mechanical stiffness, while HYDR1, with the lowest Ca^2+^ content, exhibits the highest swelling ratio and significantly lower stiffness.

**Figure 5:**
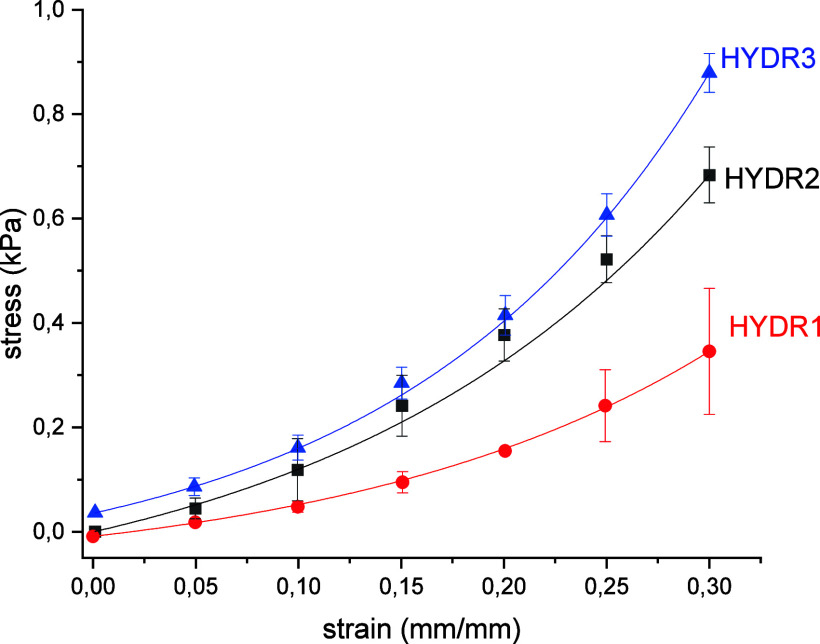
Stress–strain curves corresponding to HYDR1, HYDR2, and HYDR3 (swollen specimens), recorded under unconfined compression.

Based on the above-mentioned experimental findings, HYDR2, demonstrating an intermediate swelling ratio and good mechanical performance, was selected for use as a seed coating in further experiments, either as a pristine HYDR coating or in combination with melatonin (Mel) employed as a biostimulant.

Thermal analysis was also carried out for HYDR2 and the HYDR2-Mel. As seen in Figure 6a, for both systems the process starts with a loss of mass up to 150 °C because of the evaporation of water. The primary stage of thermal degradation occurs at 170–300 °C, and it is attributed to end-chain or backbone degradation of the alginate, which also may involve decarboxylation and depolymerization events. The HeatFlow thermograms (Figure 6b) clearly indicate endothermic transitions at 124.4 and 129.0 °C for HYDR2 and HYDR2-Mel, respectively. The addition of melatonin leads to the appearance of a sharper and slightly higher melting temperature, which may be attributed to the melting of crystalline melatonin or the existence of interactions among melatonin and the hydrogel. Bialik-Was et al.^56^ investigated the thermal properties of sodium alginate/poly­(vinyl alcohol)-based hydrogels. Their differential scanning calorimetry (DSC) analysis identified several endothermic transitions, such as loss of bound water and structural rearrangements within the hydrogel matrix. However, the thermal transitions in HYDR2-Mel are not simply melting but involve overlapping dehydration and matrix-related relaxations. In this case, the melting signal of melatonin is partially obscured by the thermal transitions of the hydrogel. Topal and co-workers^57^ presented the DSC thermogram of pure melatonin, which exhibited a distinct endothermic melting peak at approximately 118 °C. However, when an inclusion complex of melatonin was formed with hydroxypropyl-β-cyclodextrin and integrated into a chitosan scaffold, the notable melting endotherm at 118 °C was entirely absent. This disappearance of the peak suggests that melatonin transitioned from a crystalline state to an amorphous or dispersed form within the polymeric matrix.

**Figure 6:**
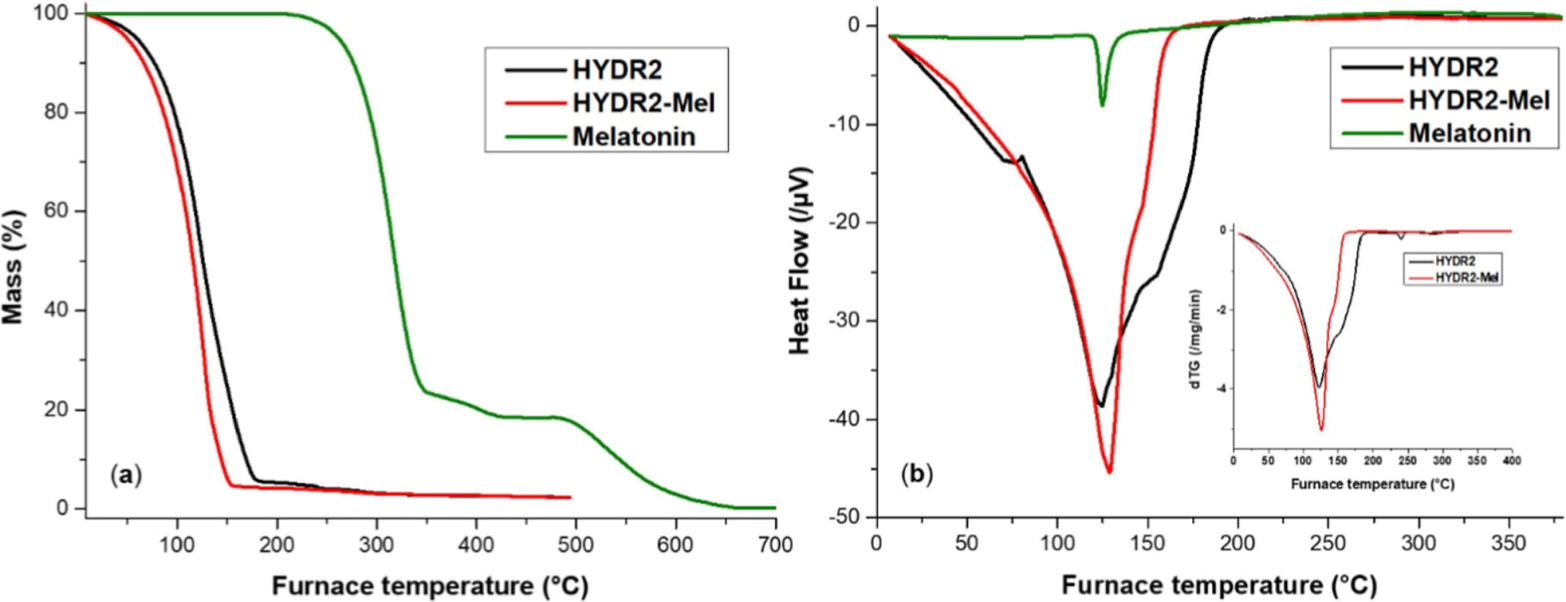
TGA (a) and HeatFlow curves with dTG shown as an inset (b) of HYDR2 and HYDR2-Mel hydrogels.

The dTG curves shown as an inset in Figure 6b confirm these trends, with distinct decomposition rate peaks becoming more intense and slightly shifted in the melatonin-loaded sample, particularly 122.7 and 126.3 °C for HYDR2 and HYDR2-Mel, respectively. These shifts suggest that melatonin influences both the thermal stability and degradation mechanism of the hydrogel, likely through physical entrapment and weak interactions with the alginate network.

### 3.2 HYDR2-Mel: Kinetic Release Studies

To investigate the release rate of Mel biostimulant encapsulated within HYDR2, kinetic studies were performed in aqueous media by means of UV–vis spectrophotometry for Mel-loaded HYDR2. As seen in Figure 7, the characteristic absorption signal of Mel appearing at 224 nm increases with time, demonstrating the sustained release of this biostimulant from the HYDR2 within 3 h.

**Figure 7:**
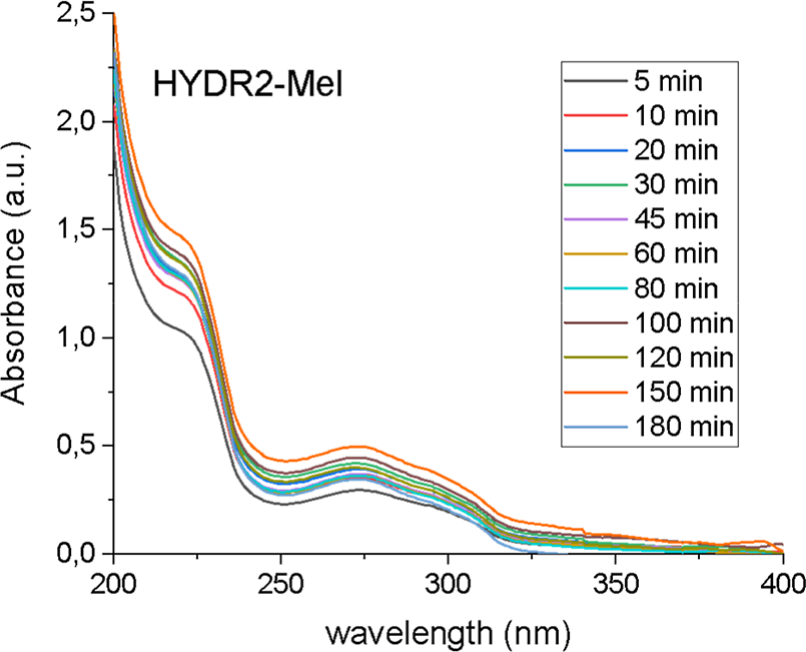
Mel release studies, performed at room temperature, upon immersing the HYDR2-Mel in DI water and recording the UV–vis spectrum of the supernatant solution at different time intervals.

### 3.3 Effects of NaCl on In Vitro Germination of Tomato Seeds

To capture the effects of the treatments on seed germination under control, 50 mM NaCl, and 75 mM NaCl, eight different germination indicators were calculated based on the daily measurements from the first successful count (3rd day) until the end of the experiment (17th day) (Figures 8 and 9).

**Figure 8:**
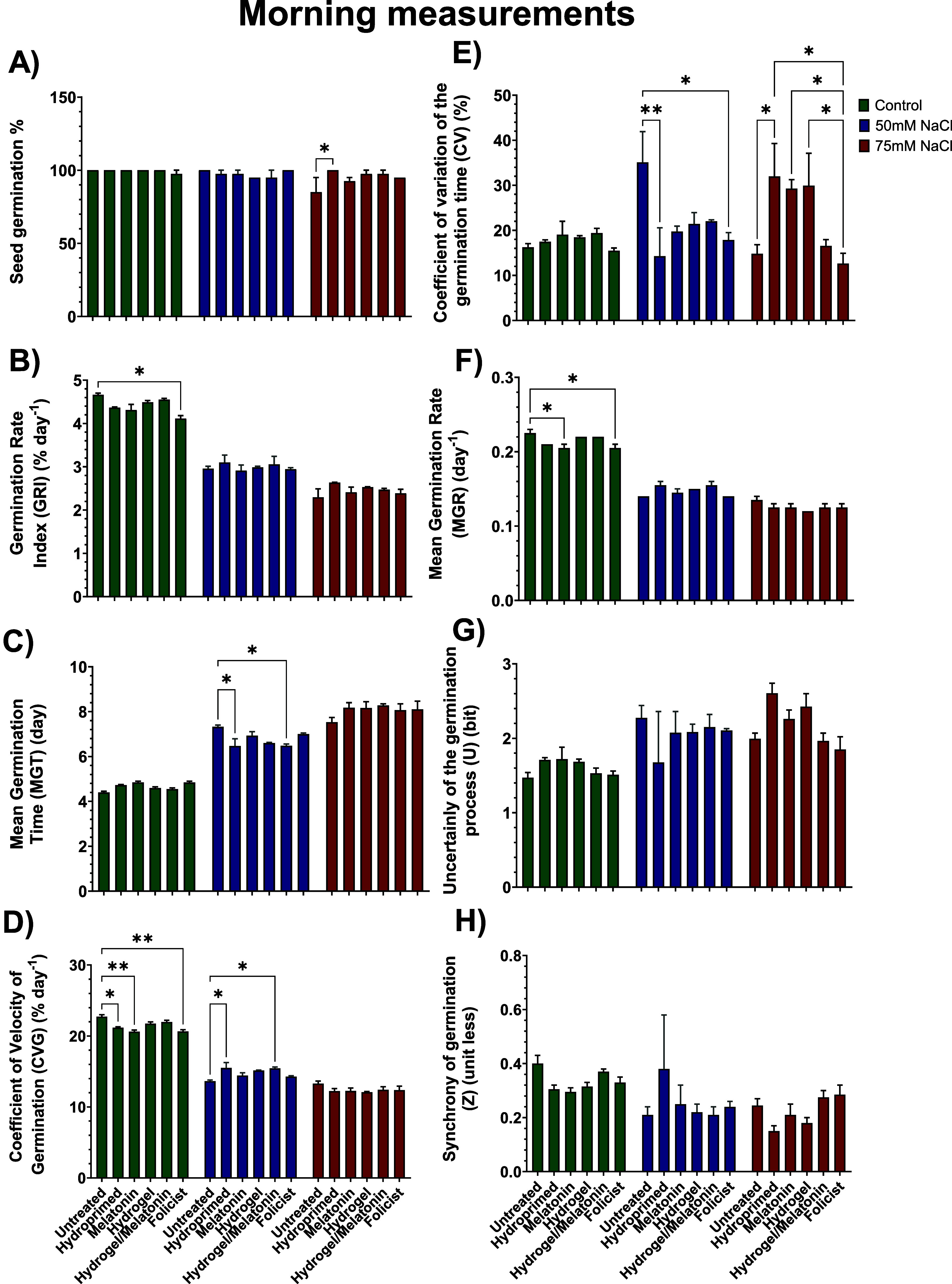
Effect of all treatments on (A) seed germination, (B) germination rate index (GRI), (C) mean germination time (MGT), (D) coefficient of velocity of germination (CVG), (E) coefficient of variation of the germination time (CV), (F) mean germination rate (MGR), (G) uncertainty of the germination process (*U*), and (H) synchrony of germination (*Z*) of morning measurements in control (0 mM NaCl), low (50 mM NaCl), and high (75 mM NaCl) salt-stressed conditions. Data are the means of three biological replications ± SE.

**Figure 9:**
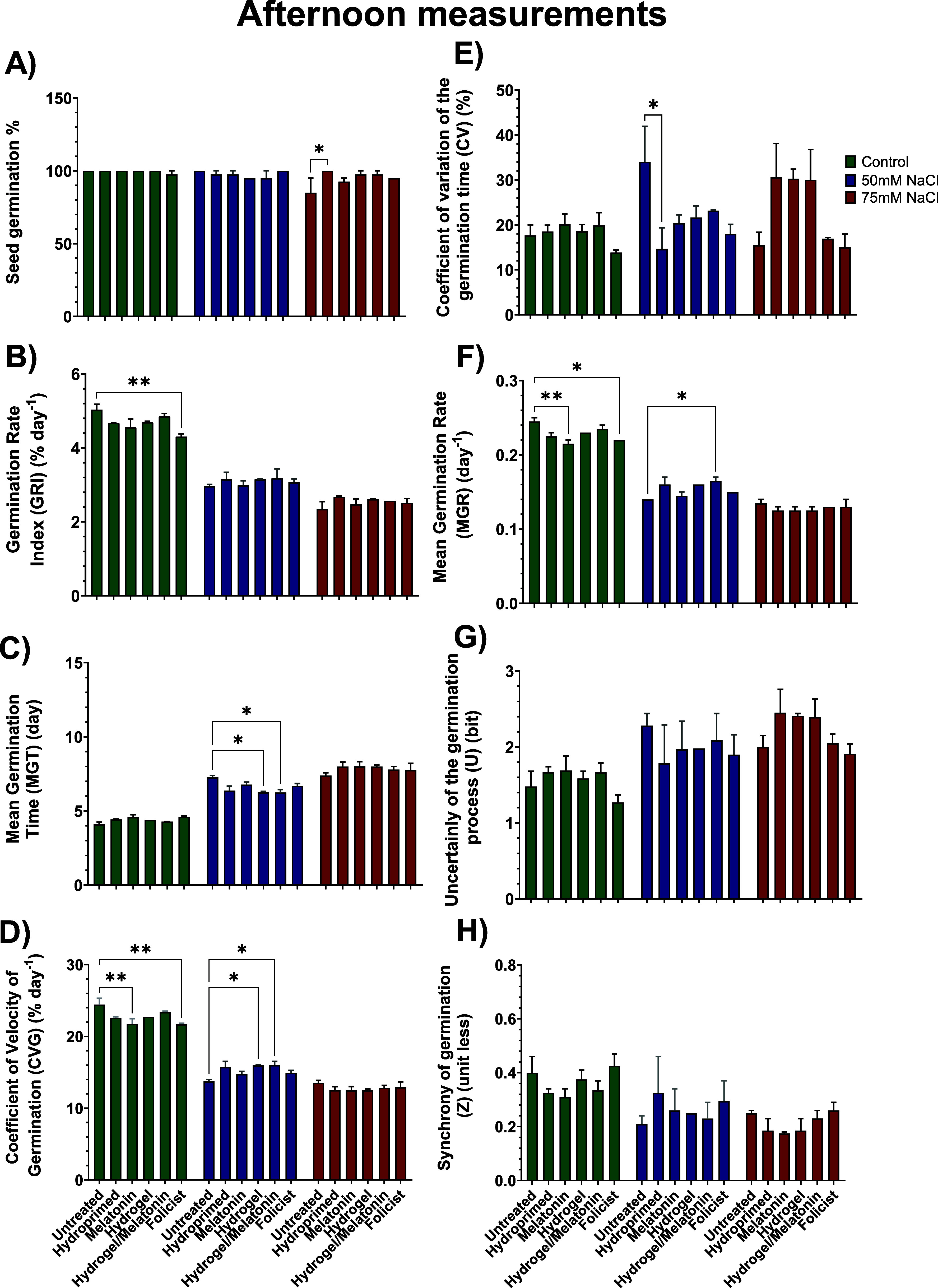
Effect of all treatments on (A) seed germination, (B) germination rate index (GRI), (C) mean germination time (MGT), (D) coefficient of velocity of germination (CVG), (E) coefficient of variation of the germination time (CV), (F) mean germination rate (MGR), (G) uncertainty of the germination process (*U*), and (H) synchrony of germination (*Z*) of afternoon measurements in control (0 mM NaCl), low (50 mM NaCl), and high (75 mM NaCl) salt-stressed conditions. Data are the means of three biological replications ± SE.

#### 3.3.1 Seed Germination % (*G*)

The seed germination % (*G*) did not reveal any major statistical differences across the three stress conditions and between the morning and evening measurements. UNT plants that grew under 75 mM NaCl were the only exception, with 85% total germination, the worst-performing group among all of the treatments (Figures 8A and 9A). Overall, seeds of all the groups germinated under well-watered conditions except FOL (97.5%). In addition, HYD and MEL seeds germinated at 97.5% and HYDR and HYDR-MEL at 95% under 50 mM NaCl. Finally, except for HYD seeds, very few seeds did not germinate in all the other treatments under 75 mM NaCl. Priming seeds generally helps them to germinate faster and in a higher percentage than nonprimed seeds (e.g., UNT), even under suboptimal conditions, because they have already completed some of the initial steps of germination.^8^

#### 3.3.2 Germination Rate Index (GRI)

The rate of germination over time was generally slightly higher in the afternoon under all treatments and stress conditions (Figures 8B and 9B). However, the main outcome was that the gradually increased salt environment negatively affected the GRI of the seeds. Overall, approximately 4.5–5% of seeds germinated at each time point under well-watered conditions, ∼3% in low salinity, and 2–2.5% in high salinity conditions. Under well-watered conditions, the FOL treatment resulted in the lowest value among all treatments, showing a statistically significant decrease of 16% compared with the UNT. Under the two salt concentrations, there were no statistically significant differences among the treatments. However, the UNT group had the lowest GRI value compared with all the other treatments.

#### 3.3.3 Mean Germination Time (MGT)

The MGT was similar in both time points across all conditions (Figures 8C and 9C). Seeds under well-watered conditions had an MGT of ∼5 days, while it was ∼7 days and ∼8 days at low and high salinity, respectively. Under the low salinity conditions, UNT seeds had the highest MGT compared with all the other treatments, while HYDR and HYDR-MEL ones had the lowest, showing the earliest germination.

#### 3.3.4 Coefficient of Velocity of Germination (CVG)

The speed of germination, as indicated by the CVG, mirrored that of the GRI (Figures 8D and 9D). The main outcome again was that the gradually increased salt environment negatively affected the CVG of the seeds. Overall, approximately ∼20% of seeds germinated under well-watered conditions, ∼15% in low salinity, and ∼12% in high salinity conditions. Under well-watered conditions, the FOL treatment resulted in the lowest value among all treatments, showing a statistically significant decrease of 13% compared with the UNT. Under low salt concentration, HYDR and HYDR-MEL CVG were statistically significantly higher compared with the UNT ones and the highest among all the treatments at the afternoon’s measurements (Figure 9D).

#### 3.3.5 Coefficient of Variation of Germination Time (CV)

The CV values, representing the uniformity of germination times, were similar at both time points (Figures 8E and 9E). No statistically significant difference was observed among the treatments under the well-watered conditions. However, under low salinity conditions, UNT seeds had the highest CV compared with all of the other treatments, indicating high uniformity in germination. HYD and FOL seeds had a statistically lower CV by 60% and 49%, respectively. As for the high salinity conditions, HYD, MEL, and HYDR seeds had statistically lower CV by 56%, 53%, and 54%, respectively, compared with the UNT ones. FOL seeds had the lowest CV among all of the other treatments.

#### 3.3.6 Mean Germination Rate (MGR)

The MGR, being inversely related to MGT, gradually declined as the salinity stress was getting higher on each occasion (Figures 8F and 9F). There were very few statistically significant differences reported. Under well-watered conditions, UNT seeds resulted in an MGR of 0.25^–1^ in the afternoon compared with MEL and FOL that resulted in 0.21^–1^ and 0.22^–1^, respectively. In contrast, UNT seeds had the lowest MGR (0.14^–1^) and HYDR-MEL the highest (0.16^–1^) under low salinity conditions (Figure 9F).

#### 3.3.7 Uncertainty of the Germination Process (*U*)

The uncertainty (*U*) was generally the same for all the treatments at both time points among all the treatments. The lack of statistically significant differences in the (*U*) across the treatments in every stress condition suggests that these factors did not significantly influence the predictability of the germination.

#### 3.3.8 Synchrony of Germination (*Z*)

The lack of statistically significant differences in the (*Z*) across the treatments in every stress condition suggests that the uniformity or coordination of seed germination was not influenced. This shows that, regardless of the treatment applied, the seeds germinated in a similarly synchronized manner.

### 3.4 Total Plant Length and Fresh Weight

To capture the effects of the treatments on plant growth under control, 50 and 75 mM NaCl, total plant fresh weight, and length were measured at the end of the experiment. Measurements of plant growth parameters showed, as expected, that the growth of stressed plants was gradually and negatively affected by increasing salinity levels (Figure 10). For example, UNT FW was lighter by 9% and 32% under 50 and 75 mM NaCl, respectively, compared with their control ones (0 mM NaCl). Furthermore, UNT plants were 22% and 35% shorter under 50 and 75 mM NaCl, respectively, compared with their controls (0 mM NaCl). These results are consistent with several other scientific studies, which have shown that these parameters, including dry weight and root growth, are inhibited under similar conditions and concentrations of salinity.^58,59^ HYDR and HYDR-MEL plants seemed to have the best performance at both growth parameters under all stress conditions. These two groups of plants had significantly higher FW compared with the UNT ones under 50 mM NaCl (by 20% and 17%), following the same trend at 75 mM NaCl (by 7% and 11%), respectively. Furthermore, the plants of the same groups were longer by 24% and 18% under low salinity and 16% and 18% under the higher concentration, respectively. On the other hand, HYDR-MEL plants demonstrated 19% higher plant height compared with UNT ones. According to a previous report, the direct addition of hydrogel to the substrate significantly increased moisture content in lettuce plants, which in turn enhanced the substrate’s water retention capacity, improving lettuce growth and fresh weight under drought stress compared with control samples.^60^

**Figure 10:**
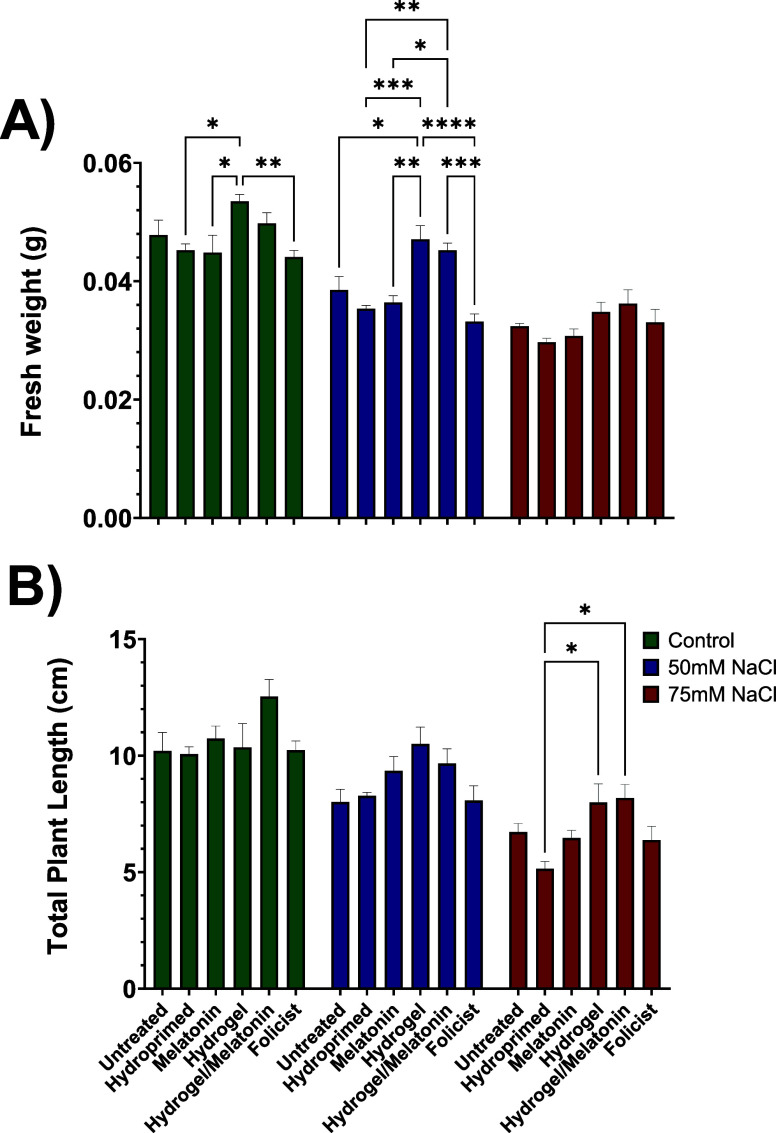
Effect of all treatments on total plant (A) fresh weight and (B) length in control (0 mM NaCl), low (50 mM NaCl), and high (75 mM NaCl) salt-stressed conditions. Data are the means of three biological replications ± SE.

Similarly, El Idrissi et al.^61^ highlighted the positive effects of hydrogel application on improving tomato plant tolerance to water deficit stress while mitigating negative impacts on various growth parameters including plant height, number of leaves, chlorophyll content, plant weights (both fresh and dry), and nutrient concentrations in different plant parts. Coating seeds with these hydrogels creates a protective microenvironment around the seed, enhancing water retention and enabling the slow release of water or priming agents (e.g., MEL). This supports a better seed establishment and stress resilience from the outset. Melatonin is reported to play a role in regulating stress-responsive mechanisms and enhancing antioxidant defenses. This priming agent could enhance growth under well-watered conditions, suggesting that it could support not only stress mitigation but also contribute to overall plant vigor and developmental robustness.^62–64^

### 3.5 Determination of Cellular Stress Markers

To evaluate the effect of seed coating treatments against oxidative stress caused by the two concentrations of salinity stress, hydrogen peroxide (H_2_O_2_) and malondialdehyde (MDA) contents were measured. These are two of the main indicators of cellular damage to the plants. H_2_O_2_ is a major reactive oxygen species (ROS) that is overproduced during stress, causing oxidative damage, whereas MDA content reflects membrane integrity and overall cell health, being produced as a byproduct of lipid peroxidation. High levels of these markers typically indicate increased oxidative stress, which can imply negative effects on plant growth and development.^65,66^ As demonstrated in Figure 11, both stress markers followed a similar pattern in most of the treatments, gradually increasing as the salt concentration increased. Tomato seedlings that were pretreated with the main important treatments, MEL, HYDR, HYDR-MEL, and FOL, had statistically lower values in both markers compared with controls (UNT, HYD) under both salinity concentrations. This indicates that these plants were less stressed overall. Among all the treatments under 50 mM NaCl, HYD-MEL and FOL plants had the lowest H_2_O_2_ value, with 6.75- and 7.2-fold decreases in content compared with the UNT ones. MEL and HYDR revealed 2- and 1.85-fold decreases, respectively. As for the H_2_O_2_ content at 75 mM NaCl, HYD-MEL had the best performance with a 6.8-fold decrease compared with the UNT ones, followed by MEL with 5.6-fold. Furthermore, FOL-treated plants showed a 4.1-fold decrease in H_2_O_2_ content, followed by HYD with a 1.5-fold decrease. Overall, MEL, HYD-MEL, and FOL plants seemed to maintain their H_2_O_2_ content at similar levels under all growth conditions compared with the other treatments.

**Figure 11:**
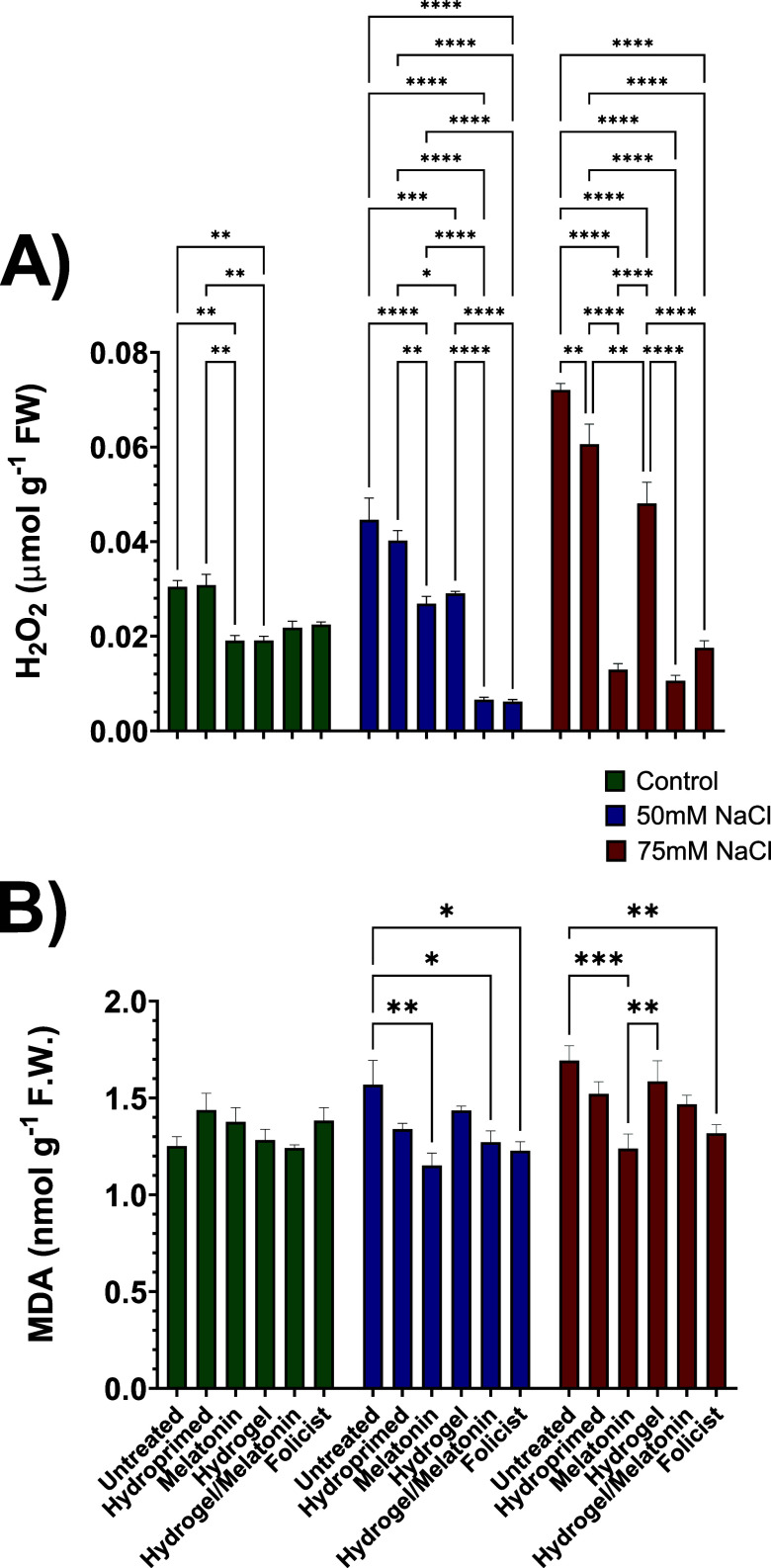
Effect of all treatments on cellular damage indicator production in control (0 mM NaCl), low (50 mM NaCl), and high (75 mM NaCl) salt-stressed conditions. (A) H_2_O_2_ and (B) MDA quantification. Data are the means of three biological replications ± SE.

These results can be explained by the ameliorative antioxidant capacity that is typically induced by priming agents like MEL or FOL that are rich in folic acid and glycine betaine.^33,67,68^ These agents activate the plant’s antioxidant defense mechanisms even before exposure to abiotic stress such as salinity. This activation includes upregulation of antioxidant enzymes like superoxide dismutase (SOD), catalase (CAT), and ascorbate peroxidase (APX), which are vital for scavenging ROS like H_2_O_2_.^33,69^ MEL is also known for its ability to work as a direct ROS scavenger.^33^ As a result, plants that are primed can prevent its accumulation in the cells. As for the MDA content, MEL-treated plants performed optimally under salt stress conditions. Other notable results include the HYD-MEL- and FOL-treated plants that revealed 1.2- and 1.24-fold decreases at low salinity and 1.15- and 1.28-fold decreases in MDA content, respectively. These lower MDA content values in the seeds pretreated with the priming agents seem to align with the similar pattern of lower H_2_O_2_ content that was observed. This suggests that despite the priming agents effectively alleviating oxidative stress, some membrane damage happened as a result of extended salinity exposure, an outcome that has been previously observed.^70^

## 4 Conclusions

The protective effect of calcium alginate-based hydrogel seed coatings containing melatonin was studied in the “Dafni F1” tomato cultivar against salinity stress under in vitro conditions. An array of agronomic and biochemical parameters was evaluated, further to detailed material characterization, with findings showing that the HYDR-Mel formulations offer significant protection against salinity-induced damage, constituting a promising “green” approach to mitigate abiotic stress effects in plants. Further studies are needed in order to reveal the *modus operandi* of the stimulatory and priming effects of hydrogel coatings with embedded biostimulants, potentially employing state-of-the-art omics platforms.
